# Encapsulation of Rat Bone Marrow Derived Mesenchymal Stem Cells in Alginate Dialdehyde/Gelatin Microbeads with and without Nanoscaled Bioactive Glass for In Vivo Bone Tissue Engineering

**DOI:** 10.3390/ma11101880

**Published:** 2018-10-01

**Authors:** Ulrike Rottensteiner-Brandl, Rainer Detsch, Bapi Sarker, Lara Lingens, Katrin Köhn, Ulrich Kneser, Anja K. Bosserhoff, Raymund E. Horch, Aldo R. Boccaccini, Andreas Arkudas

**Affiliations:** 1 Department of Plastic and Hand Surgery, University Hospital Erlangen, Friedrich-Alexander-University Erlangen-Nuremberg, 91054 Erlangen, Germany; ulrike.rottensteiner@fau.de (U.R.-B.); lara.f.lingens@studium.uni-erlangen.de (L.L.); KatrinKoehn@web.de (K.K.); ulrich.kneser@bgu-ludwigshafen.de (U.K.); raymund.horch@uk-erlangen.de (R.E.H.); 2 Institute of Biochemistry, Friedrich-Alexander-University Erlangen-Nuremberg, 91054 Erlangen, Germany; anja.bosserhoff@fau.de; 3 Institute of Biomaterials, Department of Materials Science and Engineering, Friedrich-Alexander-University Erlangen-Nuremberg, 91058 Erlangen, Germany; rainer.detsch@ww.uni-erlangen.de (R.D.); bapisarker@daad-alumni.de (B.S.); 4 Department of Hand-, Plastic- and Reconstructive Surgery—Burn Center, BG Trauma Center Ludwigshafen, University of Heidelberg, 67071 Ludwigshafen, Germany

**Keywords:** alginate dialdehyde, gelatin, nanoparticles, bioactive glass, tissue engineering, mesenchymal stem cells

## Abstract

Alginate dialdehyde (ADA), gelatin, and nano-scaled bioactive glass (nBG) particles are being currently investigated for their potential use as three-dimensional scaffolding materials for bone tissue engineering. ADA and gelatin provide a three-dimensional scaffold with properties supporting cell adhesion and proliferation. Combined with nanocristalline BG, this composition closely mimics the mineral phase of bone. In the present study, rat bone marrow derived mesenchymal stem cells (MSCs), commonly used as an osteogenic cell source, were evaluated after encapsulation into ADA-gelatin hydrogel with and without nBG. High cell survival was found in vitro for up to 28 days with or without addition of nBG assessed by calcein staining, proving the cell-friendly encapsulation process. After subcutaneous implantation into rats, survival was assessed by DAPI/TUNEL fluorescence staining. Hematoxylin-eosin staining and immunohistochemical staining for the macrophage marker ED1 (CD68) and the endothelial cell marker lectin were used to evaluate immune reaction and vascularization. After in vivo implantation, high cell survival was found after 1 week, with a notable decrease after 4 weeks. Immune reaction was very mild, proving the biocompatibility of the material. Angiogenesis in implanted constructs was significantly improved by cell encapsulation, compared to cell-free beads, as the implanted MSCs were able to attract endothelial cells. Constructs with nBG showed higher numbers of vital MSCs and lectin positive endothelial cells, thus showing a higher degree of angiogenesis, although this difference was not significant. These results support the use of ADA/gelatin/nBG as a scaffold and of MSCs as a source of osteogenic cells for bone tissue engineering. Future studies should however improve long term cell survival and focus on differentiation potential of encapsulated cells in vivo.

## 1. Introduction

Hydrogels are currently strongly investigated materials for their use as scaffolding matrices in bone tissue engineering [1]. The ability to form hydrogels in the presence of calcium ions, coupled with the possibility of being easily modulated into various shapes, and the good in vitro and in vivo biocompatibility, low toxicity, and low price make alginate a promising candidate for tissue engineering applications [2,3,4,5]. However, this polysaccharide exhibits very poor cell adhesive properties due to the absence of a cell binding ligand [6]. Addition of gelatin to alginate hydrogel has been shown to promote cell adhesion and proliferation in vitro [7,8], as well as to provide a higher viscosity of the hydrogel, resulting in improved properties for three-dimensional tissue engineering applications [3]. Although gelatin can be easily degraded in the body, alginate hydrogels do not degrade in vivo since mammals do not possess enzymes for degradation of alginate [9,10]. Oxidation of alginate allows for hydrolytic in vivo degradation of the hydrogel, and the resulting molecules are easily eliminated by the kidney [11]. Moreover, oxidation of alginate creates reactive aldehyde functional groups in the backbone of alginate that form intermolecular covalent bonds with ε-amino groups of lysine or hydroxylysine of gelatin [12].

Nano-scaled bioactive glass particles (nBG) have been combined with various biopolymers, such as alginate and gelatin [13,14]. Within biodegradable polymers, the nano-sized bioglass resembles the nano-features of the mineral particles in bone, ameliorating cell adhesion and proliferation of osteogenic cells [15,16]. The higher specific surface leads to faster solubility, higher protein adsorption, and therefore higher bioactivity than larger particles [17]. The combination of gelatin and oxidized alginate (alginate dialdehyde, ADA) with or without nBG has in general shown good biocompatibility and degradation properties both in vitro and in vivo in a recent study [18].

Delivery of osteogenic cells into a bone defect is a commonly applied technique in bone tissue engineering. Several cell types have been encapsulated into alginate and/or gelatin-based hydrogels, such as bone marrow derived mesenchymal stem cells (bmMSCs), osteoblast-like cells, or embryonic stem cells [6,19,20]. BmMSCs have a high proliferation capacity, with more than 50 cell doublings in vitro [21], and grow under relatively easy culture conditions. Both rat and human bmMSCs show a high osteogenic differentiation capacity [22,23]. Furthermore, they show immunomodulatory and pro-angiogenic properties, which could be of particular interest in bone tissue engineering [24,25].

Encapsulation of bmMSCs using a combination of gelatin and alginate dialdehyde with nBG has not been investigated yet. The goal of the present study was to evaluate survival of encapsulated cells in a specific alginate dialdehyde-gelatine based hydrogels comparing capsules with and without nBG in vitro and in vivo. Furthermore, the influence of encapsulated cells on biocompatibility, inflammation and vascularization was addressed in this preliminary study for application in bone tissue engineering.

## 2. Materials and Methods

### 2.1. Cell Isolation and Culture

Rat bone marrow derived mesenchymal stem cells (rMSCs) were isolated from adult male Lewis rats as described before [18]. The procedure was approved by the government of Middle Franconia and the according Animal Protection Committee (Az 54-2532.1-24/09). Briefly, animals were sacrificed under deep isoflurane anesthesia, and both femora and tibiae were aseptically harvested. The bone marrow cavity was flushed with 15 mL MACS buffer (PBS, Biochrom, Berlin, Germany; +2% fetal calf serum/FCS, Biochrom), and the resulting cell suspension was centrifuged at 1400 rpm for 4 min. The cell pellet was resuspended in growth medium (DMEM + Ham’s F12 1:1, Biochrom; 20% FCS, Biochrom; 1% l-Glutamine, Gibco, Darmstadt, Germany), filtered through a 100 μm pore size cell strainer (BD Biosciences, Heidelberg, Germany) and subjected to a density gradient centrifugation (Histo-Paque 1077, Sigma Aldrich, Seelze, Germany). Cells were seeded on a standard plastic 24-well plate (Greiner Bio-One, Frickenhausen, Germany) at a density of 2 × 10^6^ viable mononuclear cells/well. After 36 h, the non-adherent cells were removed by washing each well with PBS. The growth medium was changed every 2–3 days. Passaging was done at 70–80% confluency using trypsin/EDTA (ethylenediaminetetraacetic acid) solution (Biochrom, Berlin, Germany).

At passage 4, after 38 days in culture, flow cytometry was performed to confirm the presence of a pure MSC culture (expression of MSC markers, <5% endothelial cell and leucocyte surface markers). Cells from a confluent T75 flask (approximately 1.5 × 10^6^ cells) were detached as for passaging and resuspended in FACS buffer (PBS, Biochrom; 2% FCS, Biochrom; 0.05% sodium azide, Sigma Aldrich, Seelze, Germany). After centrifugation, cells were suspended in antibody solutions and incubated for 30 min. The following antibodies were used: MSC markers—CD90 FITC (BD Biosciences, clone OX-7, mouse anti rat IgG1κ); CD29 PE (eBioscience, Frankfurt, Germany; clone eBioHMb1-1, Hamster Anti-Mouse/Rat IgG); CD73 PE (BD Biosciences; mouse anti rat IgG1κ, clone 5F/B9 + rat anti mouse IgG1κ PE, BD Biosciences, clone A85-1); CD54 PerCP (eBioscience, clone 1A29, mouse anti rat IgG1κ); leucocyte marker—CD45 APC (eBioscience, clone OX-1, mouse anti rat IgG1κ); endothelial cell marker—CD31 PE (BD Biosciences, mouse anti rat IgG1κ, clone TLD-3A12); and the respective isotype controls. After incubation, cells were washed with FACS buffer and analyzed immediately (FACScalibur ^®^, BD Bioscience and FlowJo Software Version 7.6.5 for Mac, Tree Star, Ashland, OR, USA). Expression of MSC markers was 98.1% for CD90, 99.9% for CD29, 99.1% for CD54 and 92.2% for CD73. A low number of cells expressed the leucocyte marker CD45 (1.32%) and the endothelial cell marker CD31 (0.34%).

### 2.2. Preparation of ADA-GEL Solution

Alginate dialdehyde-gelatin (ADA-GEL) beads were prepared at the Institute of Biomaterials, FAU Nuremberg-Erlangen as described elsewhere [12]. Briefly, sodium alginate derived from brown algae with a guluronic acid content of 65–70% (Sigma-Aldrich, Seelze, Germany) was oxidized using sodium metaperiodate (VWR International, Leuven, Belgium) in a 1:1 ethanol-water mixture. After 6 h, ethylene glycol (VWR International) was added to stop the reaction, and the solution was dialyzed against ultrapure water (Direct-Q^®^, Merck Millipore, Darmstadt, Germany) using a dialysis membrane (MWCO: 6000–8000 Da, Spectrum Lab, Rancho Dominguez, CA, USA) for 7 days to remove unreacted periodate. After this, the solution was frozen and lyophilized. The lyophilized ADA was dissolved in PBS at a concentration of 5% (*w*/*v*). For the preparation of gelatin solution, gelatin powder from porcine skin (Bloom 300, Type A, Sigma-Aldrich, Seelze, Germany) was dissolved in ultrapure water at 37 °C at a concentration of 5% (*w*/*v*). ADA and gelatin solution were filtered through 0.45 μm and 0.22 μm filters (Carl Roth GmbH + Co. KG, Karlsruhe, Germany) respectively, prior to mixing. Sterile ADA and gelatin were mixed in an equal volume ratio by slowly adding the gelatin solution into the ADA solution under continuous stirring to facilitate homogenous crosslinking.

### 2.3. Addition of nBG and rMSCs to ADA-GEL

Nano-scale glass beads were added to the ADA solution in the respective groups (1W_ADA-GEL-rMSC-nBG and 4W_ADA-GEL-rMSC-nBG, see Table 1) prior to mixing with gelatin at a final concentration of 0.1% (*w*/*v*). Sterilization of nBG particles was achieved by dry heat sterilization technique. The composition of the nanosized bioactive glass was 47.8 wt % SiO_2_, 25.1 wt % CaO, 22.6 wt% Na_2_O, and 4.6 wt % P_2_O_5_. nBG was synthesized by flame pyrolysis and received as a gift from ETH (Zurich, Switzerland); the particle size of nBG was in the range of 20–60 nm [17,26].

rMSCs (passage 7) were detached immediately prior to encapsulation and counted using trypan blue staining (Sigma Aldrich, Seelze, Germany) and a Neubauer chamber. To allow for tracking of the rMSCs after subcutaneous implantation, cells were labeled with red fluorescent DiI (DiI cell tracker, Life Technologies, Darmstadt, Germany). 3 × 10^6^ cells were incubated for 15 min in 5 mL growth medium containing 25 µg of DiI. Cells were washed twice with PBS and then mixed with the prepared ADA-GEL(+/−nBG) solution at a final concentration of 2 × 10^6^ cells/mL of hydrogel.

### 2.4. Encapsulation

Microbeads were prepared immediately before subcutaneous implantation by pneumatic extrusion technique [12]. For this, an extrusion cartridge (Nordson EFD, East Providence, RI, USA) connected to a high precision fluid dispenser (Ultimus V, Nordson EFD, East Providence, RI, USA) was used. Microbeads were generated by applying different air pressure (2.3 bars to 2.5 bars), collected in a beaker with sterile CaCl_2_ solution (0.1 M; VWR International) and incubated for 10 min to allow for ionic gelation. The microbeads were then sieved and washed three times with serum-free DMEM to remove calcium chloride solution.

### 2.5. In Vitro Analysis

To assess the viability of encapsulated cells, live/dead staining was performed using calcein AM (Fluka Analytical/Sigma Aldrich, Seelze, Germany) for the live cells and propidium iodide (PI; Sigma-Aldrich, Seelze, Germany) for the dead cells. Cells were incubated for 7 days before the live-dead staining was performed. The staining mixture was prepared by mixing calcein AM and PI in cell culture medium at a concentration of 10 mg/mL and 1 mg/mL, respectively. The images of calcein stained cells were taken by use of a fluorescence microscope (FM) (Axio Scope A.1, Carl Zeiss Microimaging GmbH, Jena, Germany). Automatic live-dead cell counting in the fluorescence images was performed using the ImageJ software (version 1.47v, National Institutes of Health, Bethesda, MD, USA). Six images of two different magnifications (10× and 20×) per samples were used to analyze the cell number by counting the green-stained cells. The entire area of the image was calculated and the result was presented as number of cells per unit area of sample.

### 2.6. In Vivo Subcutaneous Implantation

In vivo procedures were approved by the government of Middle Franconia and the according Animal Protection Committee (Az 54-2532.1-24/09). Six adult, healthy male Lewis rats (weight 296–319 g) were used. Animals were allowed to acclimatize in the housing facility for one week prior to implantation. Rats were housed in a standardized environment (20–22 °C, RH 46–48%, light/dark cycle of 12 h) with ad libitum access to water and standard chow (Ssniff, Soest, Germany).

Two chambers filled with ADA-GEL-rMSC microbeads and two chambers filled with ADA-GEL-rMSC-nBG microbeads were inserted subcutaneously in each rat (see Table 1). The interval until explantation was 1 week (3 animals) and 4 weeks (3 animals). Custom made Teflon chambers (inner diameter 8 mm, height 6 mm, volume approx. 300 μL) were used. The weight of each chamber filled with microbeads was approximately 300 mg. Chambers were protected from light until subcutaneous implantation to avoid bleaching of the DiI staining.

Surgical procedures were performed as described in a previous study [18]. Isoflurane (Isofluran, CP Pharma, Burgdorf, Germany) was used for anesthetizing the animals, and analgesia was achieved using 0.03 mg/kg buprenorphin (Temgesic, RB Pharmaceuticals, Mannheim, Germany). A single administration of procain penicillin (22,000 IU/kg) and dihydrostreptomycin (50 mg/kg) (Veracin compositum, Albrecht, Aulendorf, Germany) was used to prevent infection of the surgical site. After aseptic preparation, two paramedian, longitudinal incisions were created on the back of each animal, and two chambers were placed on each side with the open side facing towards the underlying muscle. Chambers were sutured to the muscle (USP 4/0 Prolene, Ethicon; Johnson & Johnson, Norderstedt, Germany) and the skin was closed (USP 4/0 Vicryl Plus, Ethicon). Buprenorphin and tramadol (Tramal, 100 mg/mL, Gruenenthal, Aachen, Germany) were used for postoperative analgesia.

### 2.7. Vessel Perfusion and Explantation

After 1 or 4 weeks, vessels were perfused with India Ink under deep general anesthesia. The abdomen of the animals was opened through a midline laparotomy and the caudal vena cava and aorta were isolated. The caudal vena cava was cannulated, the aorta was severed and the vessels were flushed with heparinized saline solution. Afterwards, a mixture of India Ink (Lefranc-Bourgeois; Colart Deutschland GmbH, Maintal, Germany; 20 mL), saline solution (0.9%; 20 mL), mannite (Carl Roth GmbH & Co. KG; Karlsruhe, Germany, 1.2 g) and gelatin (Carl Roth GmbH & Co. KG; 1.5 g) was injected. The mixture was allowed to cure at −20 °C for 2 h. Subsequently, chambers were transferred to 4% formaldehyde solution (Histofix 4%, Carl Roth GmbH & Co. KG, Karlsruhe, Germany) for 24 h and then processed for histology.

### 2.8. Histological Processing

Three-micrometer cross sections were cut in two standardized planes (1 mm left and 1 mm right of the central plane). Hematoxylin-eosin (H&E) staining was done following standard protocols. ED1 (detection of macrophages/immune reaction) and Lectin (Bandeiraea Simplicifolia agglutinin, BS-1; detection of endothelial cells) were evaluated using immunohistochemistry as described elsewhere [18]. For assessment of cell survival in fluorescence microscopy, DAPI and TUNEL stainings were performed.

Lectin: Paraffinated sections were deparaffinated, cooked in citrate buffer (pH 6) for 1 min at 121 °C and transferred to 3% H_2_O_2_ (10 min) (Merck, Darmstadt, Germany). Subsequently, sections were incubated in an avidin and biotin blocking solution (Avidin/Biotin Blocking Kit, Vector Labs; Biozol, Eching, Germany). Goat serum (Vector Labs) 10% was applied for 30 min. Slides were incubated overnight in the fridge in biotinylated Lectin antibody (Isolectin B4 Biotin labeled, Sigma Aldrich, Munich, Germany; 1:270 in TRIS buffer). Detection of isolectin binding was done with a streptavidin AB Complex/HRP (Dako GmbH, Hamburg, Germany), followed by development with DAB+ chromogen (Dako GmbH, Hamburg, Germany) and counterstaining with hematoxylin solution.

ED1 (CD68): ED1 binds to the rat homologue of human CD68, a 90–110 kDa protein found on macrophages and monocytes [27]. Slides were deparaffinzed and cooked at 121 °C for 1 min. After application of a blocking solution (Zytochem Plus AP Polymer Kit, Zytomed Systems, Berlin, Germany), slides were incubated overnight in the fridge in a primary antibody solution (monoclonal mouse anti-rat CD68, Serotec, Düsseldorf, Germany; 1:300). On the following day, an enhancement reagent (Zytochem Plus AP Polymer Kit) was applied, followed by an AP-coupled secondary antibody (anti-mouse/anti-rabbit, 30 min) and development with a Fast Red solution. Counterstaining was done with hemalaun solution.

TUNEL and DAPI staining: A TUNEL assay (Terminal deoxynucleotidyl transferase dUTP nick end labeling) was used to detect DNA fragmentation and thus apoptosis in implanted rMSCs. A commercially available kit (FragELTM DNA Fragmentation Detection Kit, Fluorescent TdT Enyzme, Calbiochem/Merck-Millipore, Darmstadt, Germany) was used for this purpose. After dewaxing, sections were covered with proteinase K solution (2 mg/mL in TRIS buffer, pH 8) and incubated for 10 min at room temperature for permeabilization. After this, TdT equilibration buffer was applied for 20 min at room temperature, followed by TdT reaction mix for 90 min at 37 °C. Counterstaining of vital cells was achieved by DAPI staining (4′,6-Diamidin-2-phenylindol). After the TUNEL assay, slides were covered with DAPI solution (Sigma Aldrich; Seelze, Germany 1:1000) for 5 min at room temperature. During and after staining, slides were kept in the dark to avoid bleaching.

### 2.9. Histological Analysis

H&E and immunohistochemical images were acquired using an Olympus IX81 microscope (Olympus, Hamburg, Germany) and digital camera under 4×, 10× or 20× magnification. Fluorescence images were acquired with a Leitz DRMBE microscope (Leica Microsystems, Wetzlar, Germany) and a Leica DFC420 camera under 100× magnification. H&E images were evaluated for shape and structure of the beads, ingrowth of surrounding tissue and signs of degradation. ED1 images were evaluated for signs of inflammation and foreign body reaction (foreign body giant cells). Lectin images were evaluated for appearance of tubular lectin positive structures (newly formed vessels), and the number of lectin positive cells was counted using a computer program with a standardized threshold (ImageJ Software, National Institutes of Health, Bethesda, MD, USA; Version 1.47v). The number of DiI positive cells (rMSCs) was counted for areas adjacent to the muscle and far from the muscle using ImageJ Software with a standardized threshold and checked for co-staining with DAPI or TUNEL.

### 2.10. Statistical Analysis

Statistical analysis was done using SPSS (version 21) and GraphPad Prism 5. Normal distribution of values was controlled by means of a Kolmogorov-Smirnov test. Based on the results of this test, the mean number of DiI positive cells/mm^2^ was compared pairwise using a Mann Whitney U test between constructs with and without nBG and between the respective 1 week and 4 weeks group. The number of DiI positive cells adjacent to the muscle (per mm^2^) was compared to the number of cells far from the muscle by means of an unpaired *t*-Test or Mann Whitney U test for each group. Lectin positive cells were compared between groups using one-way analysis of variance (ANOVA) and Bonferroni’s post hoc comparison. Lectin counts from slides from a previous study with the same study design without encapsulated rMSCs [18] were used for comparison. *p*-values ≤ 0.05 were considered statistically significant. Values are given as mean ± standard deviation.

## 3. Results

rMSCs showed significant in vitro and in vivo survival after encapsulation.

**Figure 1:**
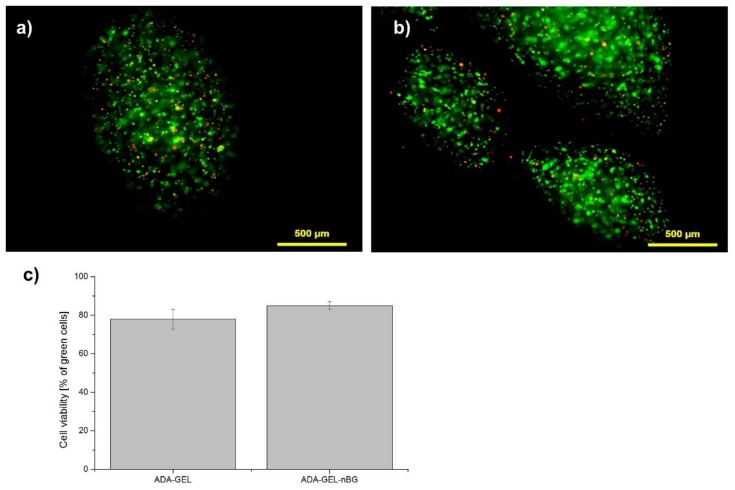
Fluorescence microscopy images of rat bone marrow derived mesenchymal stem cells (rMSCs) cells encapsulated in alginate dialdehyde-gelatin (ADA-GEL) (**a**) and ADA-GEL-nano-scaled bioactive glass (nBG), (**b**) after encapsulation (**a**,**b**: green—calcein-stained live cells in images, red—PI-stained dead cells; cells were incubated for 7 days before live-dead staining was performed). Quantitative analysis of cell viablity in ADA-GEL and ADA-GEL-nBG hydrogels, performed by image analysis (**c**).

After cell-encapsulation into the ADA-GEL and ADA-GEL-nBG microbeads, in vitro fluorescent analyses demonstrated a high percentage of vital cells with a round morphology and an even distribution throughout the beads, as seen in Figure 1 and Figure S1. The small number of dead cells (red) compared with live cells (green) confirmed that none of the materials exhibited cytotoxicity. A higher number of vital cells was found in ADA-GEL-nBG (85% green cells) compared to ADA-GEL (75% green cells), although the difference was not significant. The fabrication of the microbeads using the pneumatic pressure of 2.3 to 2.5 bars did not seem to decrease the viability of rMSC cells. Cultivation of cells after encapsulation for up to 28 days was performed with excellent results, seen in Figures S2–S4.

**Figure 2:**
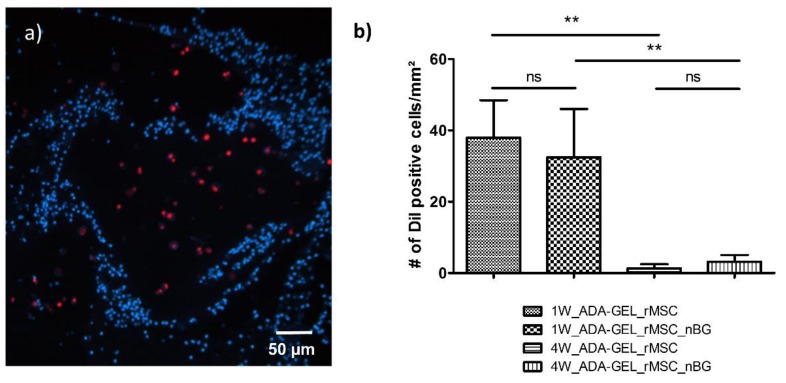
(**a**) Representative image 1 week after implantation (1W_ADA-GEL_rMSC). DiI (DiI cell tracker, Life Technologies, Darmstadt, Germany) -labeled rMSCs (red) are easily detectable within beads, and their nuclei are clearly co-stained with DAPI (blue), proving their viability; (**b**) Mean total number of DiI positive cells per mm^2^ (mean ± SD). A marked decrease was noted after 4 weeks, compared to cell survival after 1 week. ns: not significant, ** *p* ≤ 0.01 (Mann Whitney U Test).

After in vivo implantation, encapsulated rMSCs showed intense red fluorescence after histological processing, shown in Figure 2a, allowing for quantitative evaluation. The mean numbers of DiI positive cells per mm^2^ 1 week after implantation were 38.00 ± 10.46 (group 1W_ADA-GEL-rMSC) and 32.49 ± 13.56 (group 1W_ADA-GEL-rMSC-nBG). The difference between these two groups was not significant (Figure 2b). The mean cross-sectional area of the constructs was 15.00 ± 5.07 mm^2^ (group 1W_ADA-GEL-rMSC) and 18.63 ± 6.87 mm^2^ (group 1W_ADA-GEL-rMSC-nBG).

Four weeks after implantation, mean numbers of DiI positive cells per mm^2^ were 1.32 ± 1.14 (group 4W_ADA-GEL-rMSC) and 3.18 ± 1.89 (group 4W_ADA-GEL-rMSC-nBG). The mean cross-sectional area of the constructs after 4 weeks was 20.64 ± 5.31 mm^2^ (group 4W_ADA-GEL-rMSC) and 19.81 ± 3.39 mm^2^ (group 4W_ADA-GEL-rMSC_nBG). The difference between constructs with and without nBG was not significant. However, the cell number for both groups after 4 weeks was significantly lower than that for the respective groups after 1 week, as seen in Figure 2b.

**Figure 3:**
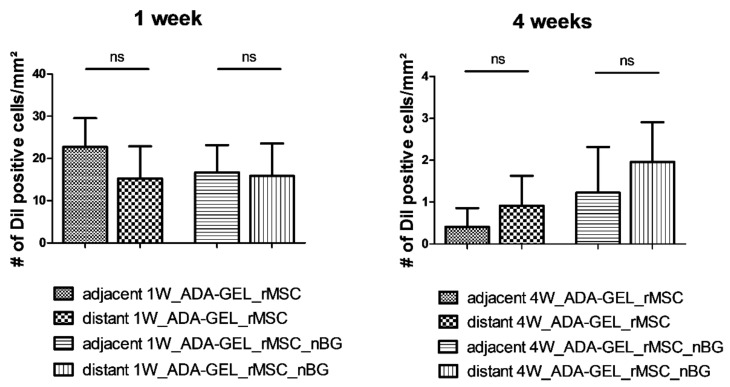
Mean number (+/− SD) of DiI positive cells per mm^2^ in different areas of the slide. Surprisingly, a higher number of DiI positive cells was present distant from the muscle after 4 weeks, compared to the areas adjacent to the muscle, although this difference was not statistically significant (ns) (Mann Whitney U Test).

After 1 week, more viable cells were present adjacent to the muscle than distant from the muscle. Surprisingly, after 4 weeks, a higher number of DiI positive cells was present in the areas distant from the muscle than adjacent to the muscle both with and without nBG, as seen in Figure 3. The differences were however not statistically significant. No DiI labeled cells could be detected in the tissue surrounding the beads in any of the constructs.

### 3.1. Tissue Integration and Beginning of Biomaterial Degradation Were Evident after In Vivo Implantation

**Figure 4:**
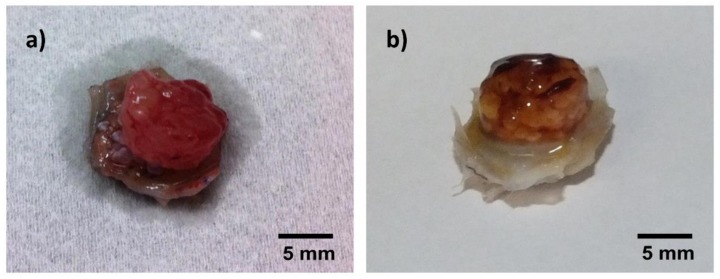

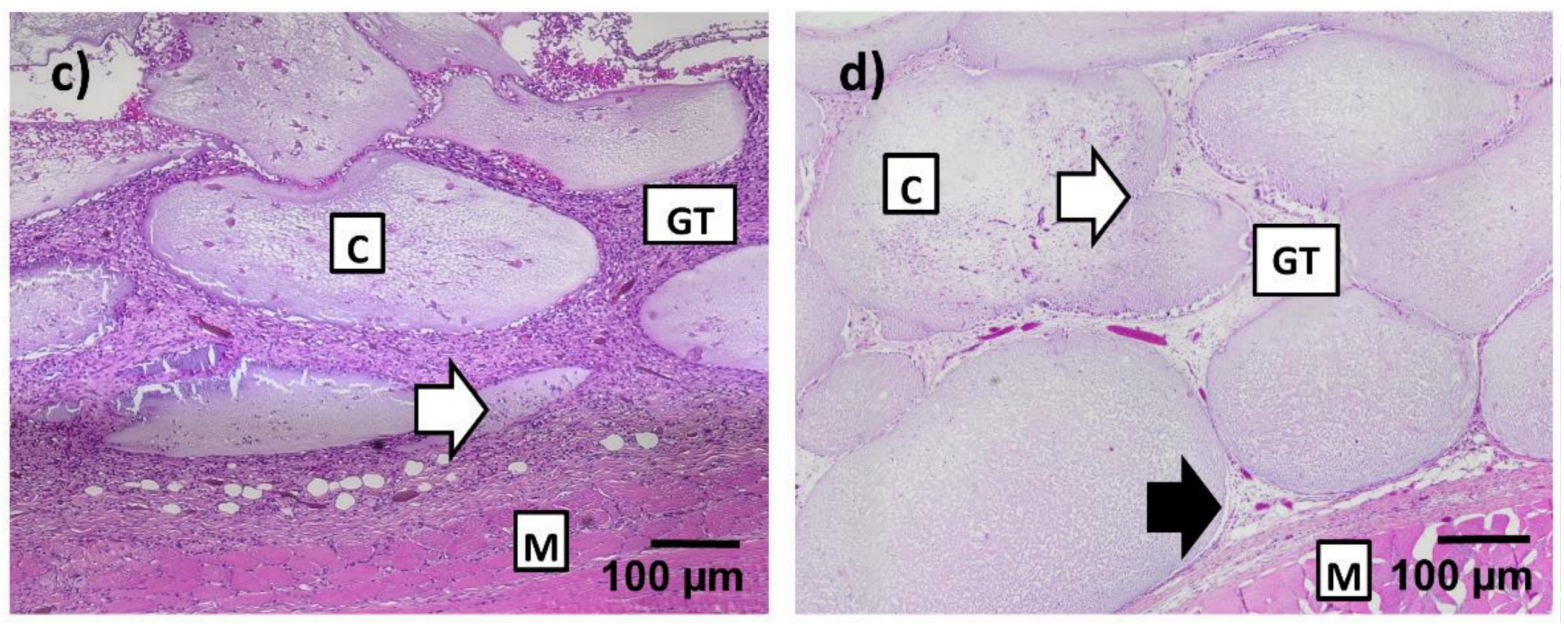
Macroscopic and microscopic (hematoxylin-eosin (H&E)-stained) appearance of constructs after explantation, representative images (not all groups are depicted, as no macroscopic difference was noticed between constructs with and without nBG). (**a**) macroscopic image, Group 1W_ADA-GEL-rMSC-nBG; loosely adhering, moist, transparent hydrogel beads; (**b**) macroscopic image, Group 4W_ADA-GEL-rMSC; firm, well adhering, opaque hydrogel beads; (**c**) H&E staining, 10×, group 1W_ADA-GEL-rMSC; loose, highly cellular granulation tissue in the areas between beads, variable degree of infiltration with small cells (arrow); (**d**) H&E staining, 10×, group 4W_ADA-GEL_rMSC; thin septae of connective tissue infiltrating the capsules (white arrow); a thin gap is visible between capsules and connective tissue (black arrow); GT = granulation tissue, C = capsules, M = muscle.

One week after in vivo implantation, the hydrogel beads were loosely adhering to each other, transparent and soft, as seen in Figure 4a. In the respective H&E staining, the beads showed a homogenous inner structure throughout the construct. Loose, highly cellular granulation tissue was present in the areas between the beads. Variable infiltration of single beads with smaller cells was visible in all constructs, with beads next to the muscle showing the highest degree of infiltration, as seen in Figure 4c.

After four weeks, beads were firm, and many of them had lost their transparency. However, they had maintained their inner structure in H&E staining throughout the construct. They were clearly adhering to the surrounding tissue and moist, as seen in Figure 4b, proving ongoing integration of the material into the surrounding tissue. Thin septae of ingrowing connective tissue could be observed into single beads in seven constructs. All beads showed variable infiltration with small, mononuclear cells. A thin gap between capsule material and the surrounding tissue could be observed, pointing to beginning degradation of the beads, as seen in Figure 4d. No difference was noted between groups with and without nBG.

### 3.2. ADA-GEL-rMSC Beads Caused a Low Immune Reaction with and without Nanoscaled BG

All animals tolerated anesthesia and surgery without any complications. The surgical procedure did not cause any notable discomfort to the animals, and all animals gained weight after surgery. A mild inflammatory reaction at the incisional site in one case and mild seroma formation around some implanted Teflon chambers were observed. At the time of explantation, two chambers appeared clearly infected and the incisions were not healed adequately (both in group 4W_ADA-GEL-rMSC). These two constructs were excluded from histological processing, resulting in n = 4 constructs in this group for final evaluation. In the remaining groups, no infection or pronounced inflammation was encountered, resulting in n = 6 constructs in each group for final evaluation.

**Figure 5:**
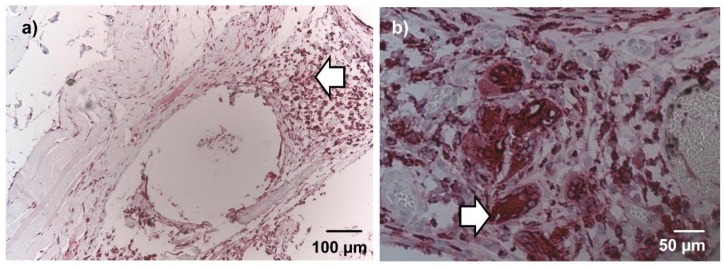
ED1 (CD68) immunohistochemical staining after 4 weeks, 10× (**a**) and 20× magnification (**b**), representative images (not all groups are depicted). A low number of ED1 positive cells was present in the granulation tissue (**a**; arrow). Few foreign body giant cells were detected in single constructs (**b**; arrow). No differences were evident between groups with nBG and without nBG in qualitative microscopic evaluation.

After 1 week, ED1-positive marcophages were diffusely dispersed in the connective tissue around the beads and the underlying muscle. Multinucleated ED1 positive cells, consistent with foreign body giant cells, were not observed at this time point. Implanted rMSCs did not show ED1 immunoreactivity. After 4 weeks, macrophages were markedly decreased and limited to the connective tissue adjacent to the beads, as seen in Figure 5a. A low number of foreign body giant cells were observed in single constructs, shown in Figure 5b. In beads with cellular infiltration, about half of the invading cells stained positive for ED1 both after 1 week and 4 weeks. No difference was noticed between groups with and without nBG.

### 3.3. Encapsulated rMSCs Promoted Vascularization through Attraction of Endothelial Cells

After one week, seven constructs appeared black due to leakage of India Ink during the perfusion process, as shown in Figure S5. After 4 weeks, pronounced leakage of India Ink was observed only in one case, proving ongoing maturation of newly formed vessels. In most recipients, the perfusion process did not lead to complete removal of erythrocytes, and the degree of India Ink perfusion was inconsistent in all groups. Therefore, counting of India Ink filled vessels was not performed for quantification of vascularization. Instead, quantification of lectin (isolectin B4)-positive cells was used as a marker of ongoing angiogenesis. According to the manufacturer, isolectin B4 binds to endothelial cells from nonprimates as well as to neurons. However, the latter structures do not form tubular structures and should be easily identifiable by their appearance in histology (lack of erythrocyte/India Ink filling in histology).

**Figure 6:**
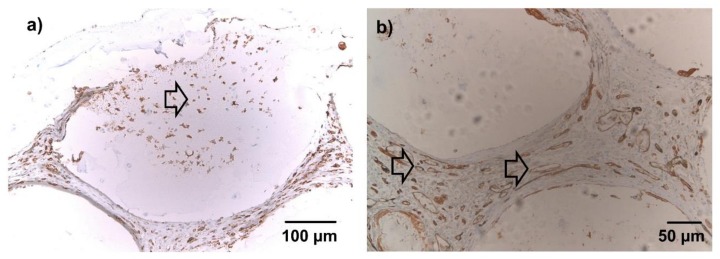
Lectin immunohistochemical staining, 10× (**a**) and 20× magnification (**b**), representative images (not all groups are depicted). Brown cells = lectin-positive; blue cells = lectin-negative (counterstained with hemalaun). (**a**) After 1 week, lectin-positive cells were diffusely dispersed in the connective tissue between beads. A fraction of the infiltrating cells stained clearly positive for lectin (arrow). (**b**) After 4 weeks, the connective tissue between the microbeads was highly vascularized (arrows). No differences were evident between groups with nBG and without nBG in qualitative microscopic evaluation.

After 1 week, lectin-positive cells were diffusely dispersed in the connective tissue between microbeads of both groups. Tubular, lectin-positive structures were not seen. In microbeads with cellular infiltration, shown in Figure 6a, a fraction of the infiltrating cells stained clearly positive for lectin. After 4 weeks, a significant number of tubular, lectin positive structures were visible throughout the constructs in both groups, most of them filled with either erythrocytes or India Ink as seen in Figure 6b. Only a small fraction of cells infiltrating the microbeads stained positive for lectin.

**Figure 7:**
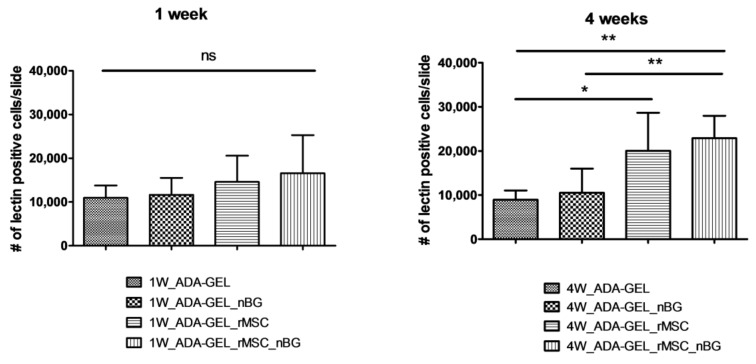
Quantitative evaluation/mean number (+/− SD) of lectin-positive cells per slide. Lectin counts from slides from a previous study with the same study design without encapsulated rMSCs [18] were used for comparison. Group 4W_ADA-GEL-rMSC showed a significantly higher number than group 4W_ADA-GEL, and group 4W_ADA-GEL-rMSC-nBG exhibited a higher number of lectin-positive cells than both groups without encapsulated cells. ns: not significant; * *p* ≤ 0.05; ** *p* ≤ 0.01 (ANOVA/Bonferroni’s Post Hoc).

The mean numbers of lectin-positive cells per slide after 1 week were 14,573.67 ± 6010.06 (group 1W_ADA-GEL-rMSC) and 16,564.42 ± 8707.57 (group 1W_ADA-GEL-rMSC-nBG). Lectin-positive cells in slides without encapsulated cells were slightly lower, counting 10,939.58 ± 2840.84 (group 1W_ADA-GEL) and 11,644.25 ± 3848.69 (group 1W_ADA-GEL-nBG). The differences between groups after 1 week were not statistically significant, as seen in Figure 7).

Lectin-positive cell numbers per slide after 4 weeks were 20,041.13 ± 8615.22 (group 4W_ADA-GEL-rMSC) and 22,886.00 ± 5098.00 (group 4W_ADA-GEL-rMSC-nBG). In groups without encapsulated cells, mean numbers per slide were 8923.17 ± 2109.81 (group 4W-ADA-GEL) and 10,542.0 ± 5459.84 (group 4W_ADA-GEL-nBG). Group 4W_ADA-GEL-rMSC showed a significantly higher number than group 4W_ADA-GEL, and group 4W_ADA-GEL-rMSC-nBG exhibited a higher number of lectin-positive cells than both groups without encapsulated cell as seen in Figure 7.

## 4. Discussion

ADA-GEL hydrogels have been successfully used for encapsulation of different cell types in vitro, including hepatocytes, MG-63 osteoblast-like cells, and adipose-derived stem cells [10,28,29]. Crosslinking of the two components can be achieved without addition of any crosslinking agents by simply mixing of the two components, which results in a Schiff’s base reaction and a covalent bonding between the ε-amino groups of lysine or hydroxylysine of gelatin and the aldehyde of ADA [10]. Obviously, this process is mild and cell friendly, making ADA-GEL a promising candidate for cell encapsulation. Furthermore, ADA-GEL hydrogel forms a porous inner structure, which supports cell proliferation [30]. The structure of ADA-GEL used in the present study was analyzed in previous studies by cryo scanning electron microscopy [30]. The hydrogel surface exhibited pore sizes of 20–1600 nm. An oxidation degree of 33% in the ADA component and a crosslinking degree of 34% were determined. Gelation time, storage moduli and degradation were analyzed respectively [12]. By adding nBG into the polymer matrix, gelation time of the hydrogels decreased, whereas the degree of crosslinking with gelatin and stiffness of the matrix increased [31].

High in vitro cell survival of rMSCs after encapsulation was found in the present study supporting the use of ADA-GEL as a promising exogenous extracellular matrix in 3D tissue engineering. The morphology of the live cells was round, which indicated that material degradation had not been started after 7 days of cultivation. This is in accordance with other studies, where cells started to spread and to migrate after 7 to 10 days of cultivation in the ADA-GEL matrix [32]. Furthermore, it should be considered that the addition of nBG further decreased the degradation rate of the hydrogel.

Only a very limited inflammatory reaction after in vivo implantation was found in the current experiment, similar to a previous study investigating ADA-GEL hydrogel without encapsulated cells [18]. The observed mild inflammation and slight seroma formation was probably due to the surgical procedure and the implanted Teflon chamber itself rather than the ADA-GEL material, as similar effects were noticed with other materials tested by our group in preliminary experiments. Both seroma and inflammatory reactions were self-limiting after 1 week. Two chambers were infected at the time of inflammation, which was caused by a small dehiscence of the suture after surgery. This led to a lower number of constructs in the group 4W_ADA-GEL_rMSC, possibly leading to the lack of statistical significance in the final evaluation of some parameters.

Bone marrow derived MSCs are widely used as a cell source in bone tissue engineering, with beneficial effects on both osteogenesis and vascularization of newly formed bone (reviewed by Roux et al. [33]). These cells have been encapsulated in a hydrogel composed of unoxidized sodium alginate and gelatin [19] and RGD-modified alginate [34] and evaluated in vivo for their bone-forming capacity. Regarding the osteogenic differentiation potential of bmMSCs in the current hydrogel composition, supportive preliminary data are available, as seen in Figure S6, and thorough evaluation will be performed in future experiments. The current study however focused on cell survival after implantation, a parameter that was not assessed in the mentioned studies. In the present work, high cell survival was seen after 1 week for the ADA-GEL hydrogels with and without nBG, which however decreased markedly after 4 weeks. A large percentage of this decrease is probably due to apoptosis of the implanted cells during this period, since no cell evasion from the beads was evident. In contrast to encapsulated cells in vitro, a lower nutrient and oxygen supply is supposed to be present in vivo where beads are not constantly surrounded by medium and enclosed in a Teflon chamber with only one open side. This suggests that cell apoptosis after in vivo implantation is inevitable to some extent due to changes in the environment of the microbeads. Cell survival was higher in beads with nBG, although this difference was not significant. This points to a high compatibility of these nanocrystalline structures and implanted MSCs in a 3D environment, which together with preliminary differentiation data, shown in Figure S6, supports further investigation of this material as a scaffold for bone tissue engineering.

In addition to decreased nutrient supply, the presence of gelatinases and other matrix-degrading enzymes in vivo and the resulting ongoing loss of gelatin from beads could have led to a decreased content in RGD-motifs in beads, which is crucial for adherent cell types such as rMSCs. Gelatinases are produced by different cell types such as neutrophils, macrophages, and fibroblasts [35,36,37] and are constantly present in plasma [38]. After one week, a variable number of cells was seen within the hydrogel beads of all constructs, with beads next to the muscle showing the highest degree of cellular infiltration. After 4 weeks, cellular infiltration was homogenous throughout the construct, likely due to vascularization that had proceeded to deeper layers. Of these infiltrating cells, a high percentage expressed ED1 (CD68), identifying them as macrophages. Other gelatinase producing cell types were not specifically evaluated by immunohistochemical staining, but might have shown the same infiltration pattern. This suggests invasion of gelatin-degrading cells originating from the muscle, were blood supply was highest. Different degrees of degradation proceeding from the muscle to deeper layers could also be the cause of higher cell survival in areas far from the muscle after 4 weeks, compared to areas adjacent to muscular tissue, possibly leading to a decrease in RGD-motifs and thus important ECM components in areas with fast gelatin degradation. In a recent study from our group, similar results were obtained in a vascularization model using a central vascular axis in a custom-made Teflon chamber [39]. In this study, encapsulated rMSCs showed higher cell survival in peripheral areas compared to areas adjacent to the vascular axis.

Vascularization is a crucial factor in bone tissue engineering, especially when aiming at large defects, when axial vascularization is often used to obtain adequate nutrient supply throughout the transplanted tissue [40]. In normal rat tissue, an average growth of newly forming vessels of 180 µm per day can be expected [41]. For the 4-weeks groups of this study, this would result in a length of 5 mm for ingrowing vessels, meaning that with a chamber height of 6 mm the deepest parts of the construct would not be vascularized before explantation. In a previous study using ADA-GEL without encapsulated cells, the resulting lack of nutrient and oxygen supply presumably led to dry microbeads and a loss of inner structure in beads deep in the chamber [18]. In the present study, clear vessel formation was detected throughout the constructs after 4 weeks, and beads in the deeper areas of the constructs had maintained their shape and inner structure. Quantification of these structures was not performed, as perfusion with India Ink led to a leakage in some constructs, impairing quantification of India Ink-filled structures. Counting of lectin-positive cells was used as a substitute for evaluating vessel number. This however should be seen as a marker of angiogenesis and not necessarily of vascularization. Encapsulation of rMSCs in the hydrogel significantly increased the number of lectin-positive cells after 4 weeks, indicating increased endothelial cell proliferation and/or invasion resulting in angiogenesis. In vitro studies showed that MG-63 cells immobilized in ADA-GEL hydrogel expressed significant levels on VEGF (vascular endothelial growth factor) [28]. Mesenchymal stem cells have shown the potential to stimulate endothelial progenitor cells by secreting proangiogenic cytokines, such as VEGF and MCP-1, both in vitro and in vivo [25,42]. Therefore, secretion of these cytokines due to ADA-GEL encapsulation could have been responsible for the high degree of angiogenesis in this experiment. Delivery of angiogenic growth factors additionally to the encapsulated cells could further increase this effect [43,44]. Our results are in accordance with Steiner et al., who showed increased vascularization originating from a central vascular axis after encapsulation of rMSCs [37]. Thus, the increase of angiogenesis in our study probably led to higher vascularization of the constructs as well, although this parameter was not quantified.

Survival of encapsulated rMSCs after 4 weeks as well as in vitro cell survival appeared slightly better in the group containing nanoscaled bioactive glass particles, although this effect was not significant. The lack of statistical significance might be attributable to the lower number of constructs in the group without nBG. Results from a previous study, in which rMSCs were seeded on ADA-GEL films for evaluation of biocompatibility suggested a cytotoxic effect of 0.1% nBG on this cell type [18]. However, this 2D environment might be different from a 3D environment as evaluated in the present study. Keshaw et al. found concentrations of 0.1% nBG to be suitable for three-dimensional cultivation in vitro [45] which is consistent with in vitro results in the present study, and the same might be true for in vivo models. In addition to better cell survival of rMSCs, the increase of lectin-positive endothelial cells appeared more pronounced in constructs containing nanoscaled bioactive glass. This is in accordance with previous in vivo studies, where BG has been shown to promote angiogenesis after subcutaneous implantation [46,47]. Furthermore, it could be demonstrated that nBG induced an increase of gene expression of VEGF-A in terminally differentiated osteoclasts, supporting a hypothesis that the combination of nBG and osteoclasts could be a trigger for angiogenesis [48]. Bioactive glass compositions similar to the one used in this study promote the release of angiogenic growth factors such as VEGF and bFGF (basic fibroblast growth factor) from fibroblasts in 2D and 3D in vitro culture [45,49,50,51,52]. A similar effect is possible for encapsulated rMSCs in vivo and should be evaluated in detail in future studies.

## 5. Conclusions

The present study shows significant in vitro as well as short-term in vivo survival of bone marrow derived MSCs encapsulated in an alginate dialdehyde/gelatin hydrogel, with a notable decrease after 4 weeks. The addition of nanoscaled bioactive glass seemed to have a positive impact on cell survival and angiogenesis. The implanted MSCs were able to attract endothelial cells and clearly promoted angiogenesis. Good biocompatibility and a low immune reaction were observed. The results of this study support the use of alginate dialdehyde/gelatin/nBG as an exogenous matrix for encapsulation of mesenchymal stem cells for bone tissue engineering.

## Figures and Tables

**Table 1:** In vivo study design. nBG: gelatin and nano-scaled bioactive glass.

Group	Encapsulated Cell Density (mL^−1^)	Concentration of nBG (*w*/*v*%)	Time until Explantation	# of Chambers
1W_ADA-GEL-rMSC	2 × 10^6^	none	1 week	6 (2 per animal)
1W_ADA-GEL-rMSC-nBG	2 × 10^6^	0.1	1 week	6 (2 per animal)
4W_ADA-GEL-rMSC	2 × 10^6^	none	4 weeks	6 (2 per animal)
4W_ADA-GEL-rMSC-nBG	2 × 10^6^	0.1	4 weeks	6 (2 per animal)

## Supplementary Materials

The following are available online at http://www.mdpi.com/1996-1944/11/10/1880/s1, Figure S1: Cell distribution in the microbeads before implantation, Figure S2: Light microscopy images of ADA-GEL and ADA-GEL nBG microbeads during 28 days of cultivation, Figure S3: Light microscopy image of rMSC in ADA-GEL nBG after 14 days, Figure S4: Fluorescence microscopy image of rMSC in ADA-Gel microbeads after 28 days, Figure S5: Pronounced leakage of India Ink after perfusion of the vascular network. Figure S6: Light microscopy images of alkaline phosphatase (ALP) stained rMSC encapsulated in ADA-GEL and ADA-GEL-nBG microbeads after four weeks of cultivation without osteogenic supplements.
