# Supplementary material for: Encapsulation of Rat Bone Marrow Derived Mesenchymal Stem Cells in Alginate Dialdehyde/Gelatin Microbeads with and without Nanoscaled Bioactive Glass for In Vivo Bone Tissue Engineering
Source: Materials (Basel). 2018 Oct 1;11(10):1880. doi: 10.3390/ma11101880 (PMC6213117; doi:10.3390/ma11101880)
Supplement: Supplementary file 1 [file materials-11-01880-s001.zip › Figure captions - supplementary material.docx]

**Figure captions - Supplementary figures**

**Figure S1**: To illustrate the cell distribution in the microbeads before implantation, Fig. S1 (FM merged with a LM image) shows rMSC cells strained with vybrant DiI. The image revealed that the rMSC were homogenous distributed in the hydrogel matrix.

LM = light microscope; FM = fluorescence microscope

**Figure S2:** LM images of ADA-GEL and ADA-GEL nBG microbeads during 28 days of cultivation. The cells were found to be uniformly distributed through the whole microbeads in all hydrogels.

**Figure S3:** LM image of rMSC in ADA-GEL nBG after 14 days. Cells were spreading, indicating substantial cell–matrix adhesions.

**Figure S4:** FM image of rMSC in ADA-Gel microbeads after 28 days. Cells were stained for the nucleus (green) and actin cytoskeleton (red). Some individual cells showed a well-spread and elongated morphology.

**Figure S5:** Pronounced leakage of India Ink was present in seven constructs of the 1-week-groups (1W_ADA_GEL_rMSC and 1W_ADA_GEL_rMSC_nBG) after perfusion of the vascular network, pointing to immature vessels. No leakage was present in 4-weeks-groups.

**Figure S6:** LM images of alkaline phosphatase (ALP) stained rMSC encapsulated in ADA-GEL and ADA-GEL-nBG microbeads after four weeks of cultivation without osteogenic supplements (above: overview and detailed image by higher magnification). Although no osteogenic supplements were applied, alkaline-phosphatase-positive cells (osteoblast-like cells) are detected, which appeared in deep red color. Measurement of specific ALP-activity showed no significant difference with or without nBG (below).
