# Supplementary figures and images for: Encapsulation of Rat Bone Marrow Derived Mesenchymal Stem Cells in Alginate Dialdehyde/Gelatin Microbeads with and without Nanoscaled Bioactive Glass for In Vivo Bone Tissue Engineering
Source: Materials (Basel). 2018 Oct 1;11(10):1880. doi: 10.3390/ma11101880 (PMC6213117; doi:10.3390/ma11101880)

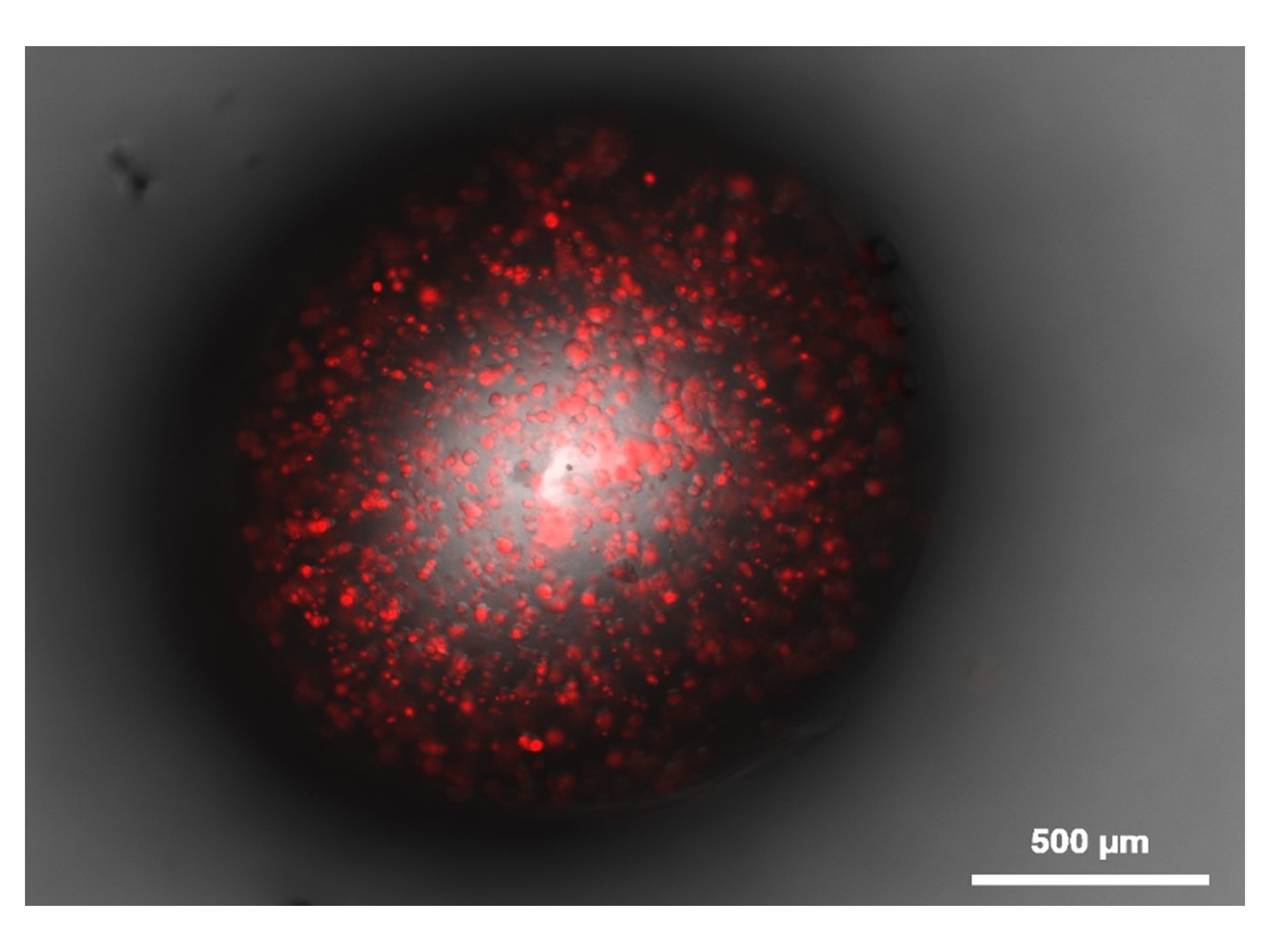

Supplement: Supplementary file 1 [file materials-11-01880-s001.zip › Fig_S1.tif]

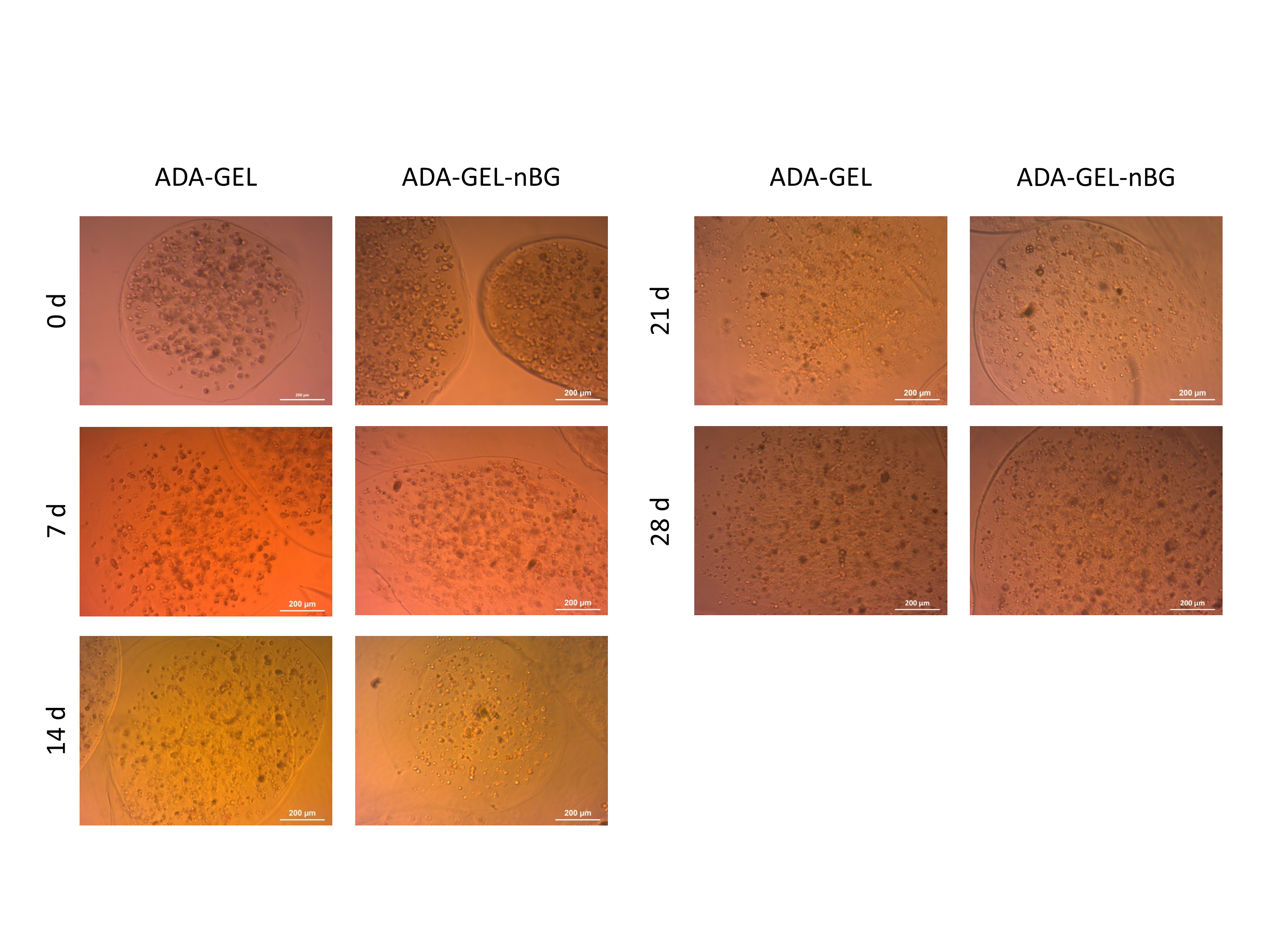

Supplement: Supplementary file 1 [file materials-11-01880-s001.zip › Fig_S2.tif]

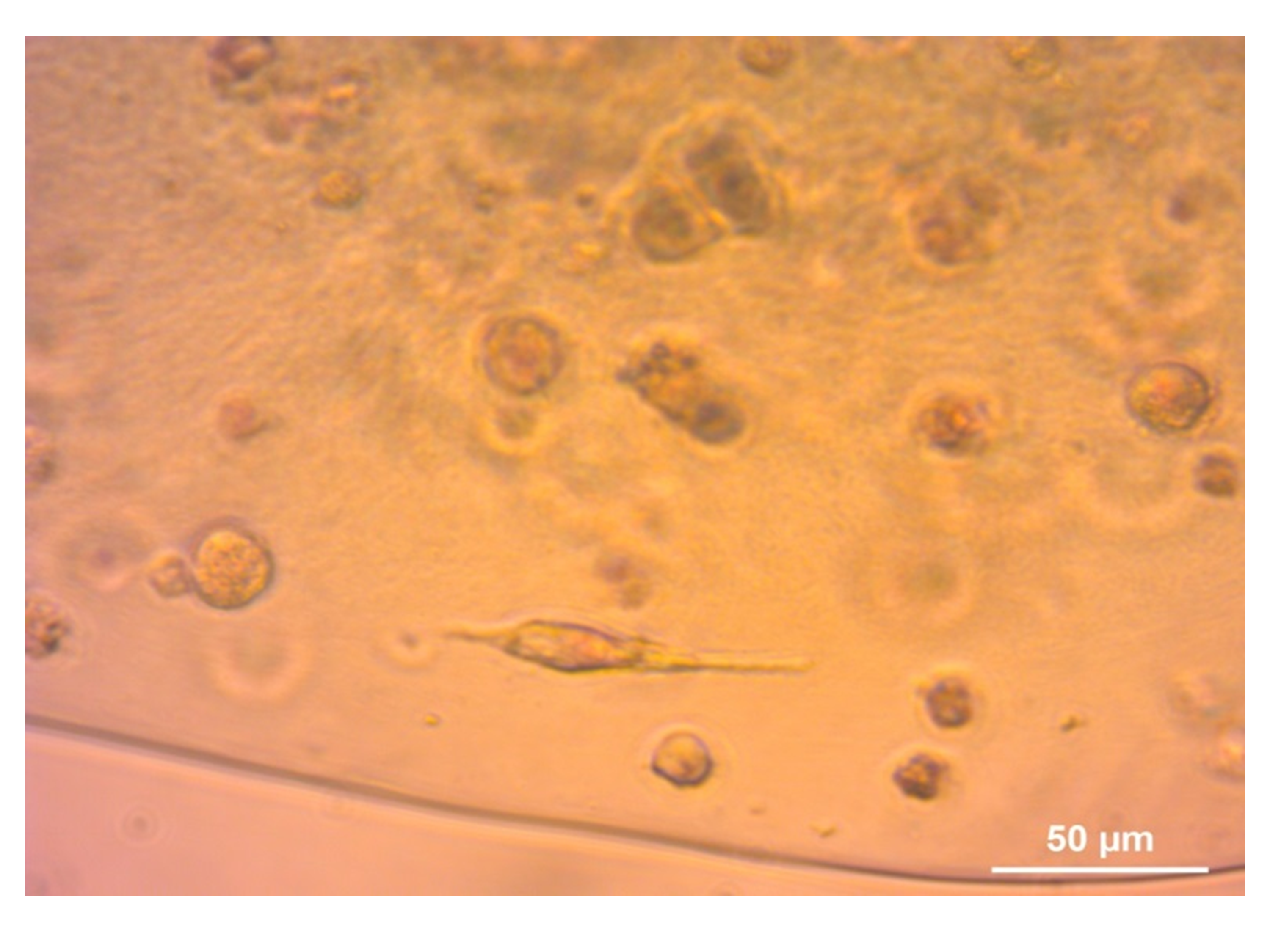

Supplement: Supplementary file 1 [file materials-11-01880-s001.zip › Fig_S3.tif]

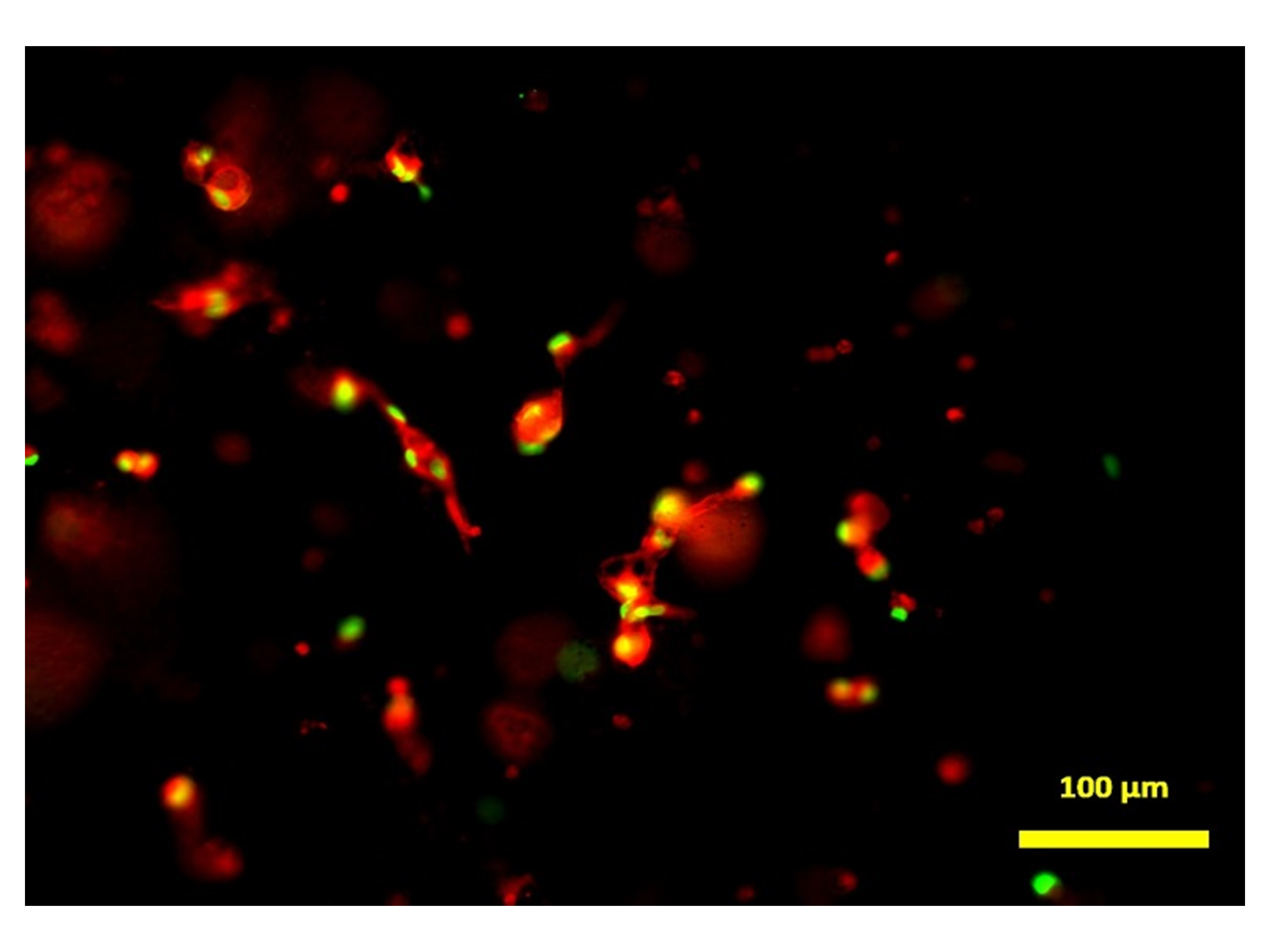

Supplement: Supplementary file 1 [file materials-11-01880-s001.zip › Fig_S4.tif]

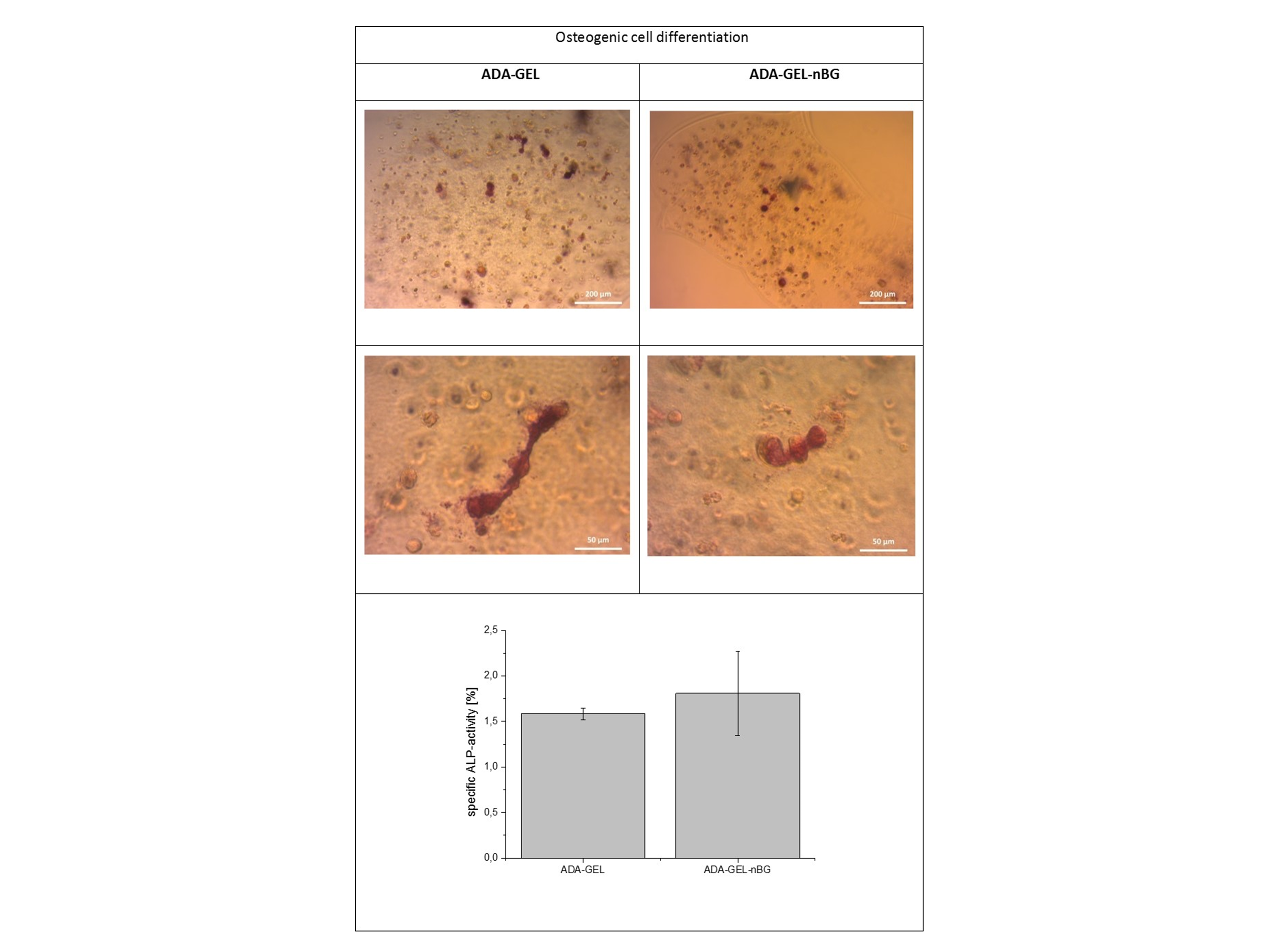

Supplement: Supplementary file 1 [file materials-11-01880-s001.zip › Fig_S6_proofread.tif]

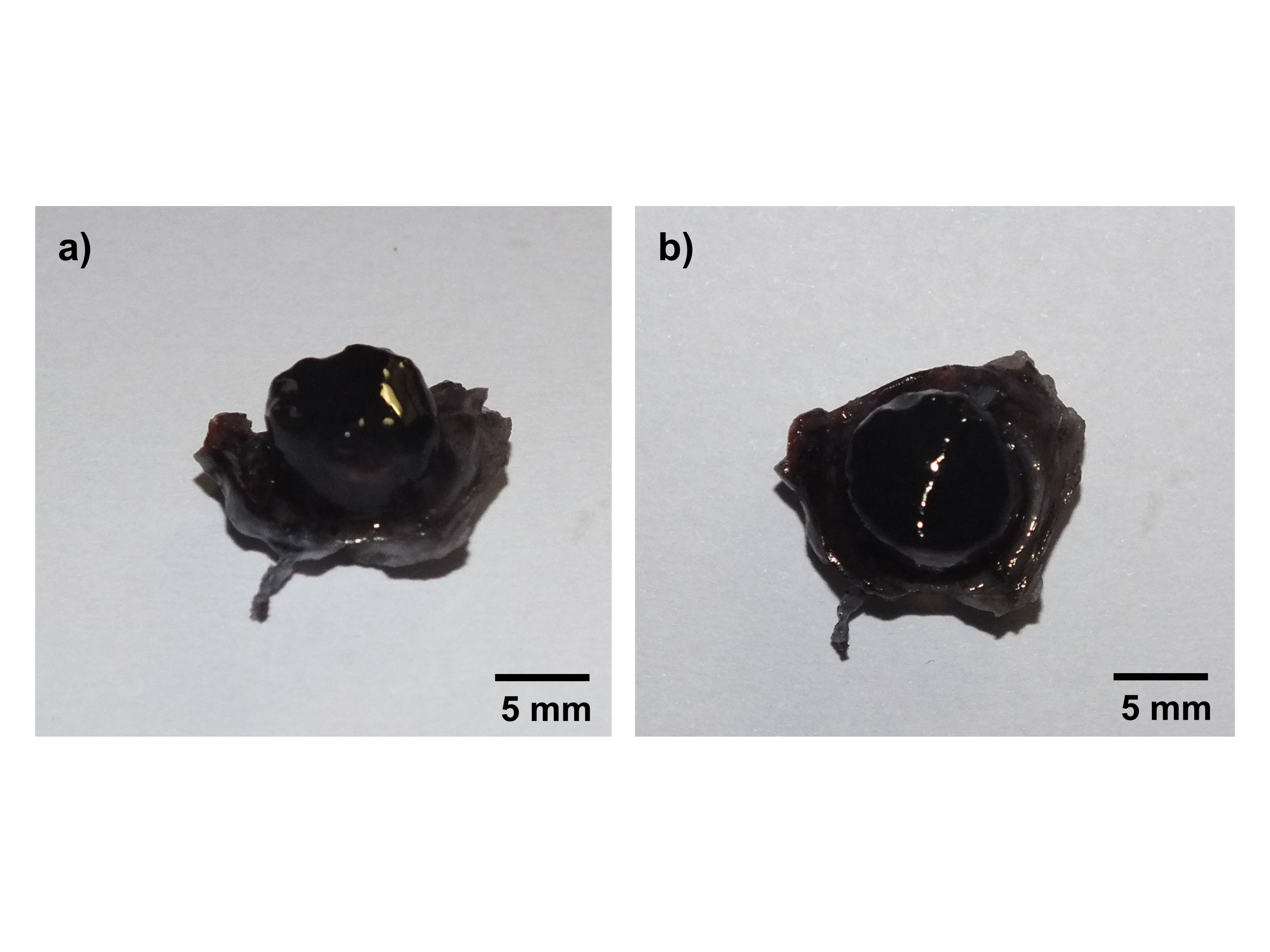

Supplement: Supplementary file 1 [file materials-11-01880-s001.zip › Fig_S5_proofread.tif]
